# Examining the benefit of L2 language proficiency on academic performance using Bayesian logistic modeling

**DOI:** 10.3389/fpsyg.2025.1613695

**Published:** 2025-10-01

**Authors:** Taocheng Jiang, ZhiYi Zheng, Feng Wang

**Affiliations:** ^1^Liberal Arts College, Hunan Normal University, Changsha, China; ^2^School of Foreign Languages, Xiangnan University, Chenzhou, China; ^3^Newcastle University, Newcastle upon Tyne, United Kingdom; ^4^School of Physical Education, Xiangnan University, Chenzhou, China

**Keywords:** L2 standard test, L2 test score, academic performance, Bayesian method, international students (foreign students)

## Abstract

**Introduction:**

The Hanyu Shuiping Kaoshi (HSK), a standardized L2 proficiency test, is a mandatory requirement for international students pursuing graduate study in China. Despite its importance, the potential benefits of higher HSK scores for predicting academic performance during the first year of graduate study remain largely unexplored.

**Methods:**

This study analyzed data from 666 graduate students enrolled in universities in southern China. The dataset included HSK listening, reading, and writing scores, as well as average undergraduate academic scores. First-year graduate academic performance was represented as a binary variable: a value of 1 indicated an average score of 85 or above (B+ or higher), while 0 indicated otherwise. A robust Bayesian logistic regression model was developed to estimate the probability of achieving good academic performance, with 80% of the dataset used for training and 20% for validation.

**Results:**

Analysis revealed statistically significant correlations among the three HSK test components. The Bayesian logistic regression model demonstrated strong predictive power in estimating the likelihood of achieving a B+ or higher in the first year of graduate school. The model effectively incorporated both L2 test scores and undergraduate academic records to generate accurate predictions.

**Discussion:**

These findings highlight the predictive value of HSK scores and prior academic achievement for international students’ success in graduate programs in China. The results suggest that L2 proficiency, as measured by the HSK, plays a meaningful role in shaping academic outcomes. This study provides evidence for universities to consider HSK performance not only as an admission requirement but also as an early indicator of students’ potential academic performance.

## 1 Introduction

Hanyu Shuiping Kaoshi, commonly abbreviated as HSK, is a standardized test of Mandarin Chinese proficiency, developed and administered by the Chinese Ministry of Education (Su and Shin, 2015; Teng, 2017; Peng et al., 2021). HSK measures Chinese language proficiency across various levels, from beginner to advanced. The test is divided into six levels, with HSK 1 and 2 targeting elementary proficiency, HSK 3 and 4 addressing intermediate skills, and HSK 5 and 6 representing advanced proficiency.

Each HSK level tests a candidate’s abilities in listening, reading, and—at higher levels—writing, which are aligned with the Common European Framework of Reference for Languages (Peng et al., 2021). The test is widely recognized by universities and employers in China and serves as an essential tool for both academic and professional advancement. For international students seeking to study at Chinese institutions, HSK is often a requirement, as it helps institutions gauge the applicant’s ability to navigate an academic setting in Mandarin. Additionally, achieving high levels on the HSK can open doors to scholarships, internships, and other opportunities within China.

### 1.1 The proficiency of L2 on academic performance

Studying a second language (L2) is crucial for students pursuing academic studies abroad in a language other than their first (Wilczewski and Alon, 2023; Campbell and Li, 2008). Language proficiency acts as the foundation for understanding and engaging with academic content, enabling students to comprehend lectures, participate in discussions, and complete assignments effectively (Yu, 2013; Andrade, 2006). Strong L2 skills empower students to access a broader range of academic resources, including textbooks, journal articles, and online materials, which are often only available in the host country’s language. Recent studies in the literature have explored how specific language skills—listening, reading, and writing—affect academic performance, particularly among international students (In’nami and Koizumi, 2021; Wallace, 2020; Vafaee and Suzuki, 2020). Listening skills, often crucial for understanding lectures and participating in classroom interactions, are frequently linked to students’ ability to follow coursework effectively (Trentman, 2013). Reading skills, essential for interpreting academic texts and conducting research, significantly impact students’ comprehension and analytical capabilities. Writing skills are similarly important, as they influence a student’s ability to express ideas clearly and adhere to academic conventions (Liu, 2024).

Beyond academic success, L2 language proficiency plays a pivotal role in facilitating cultural adaptation, which indirectly impacts academic outcomes (Cao et al., 2016; Smith and Khawaja, 2011; Jing et al., 2020; Marginson, 2014; Young and Schartner, 2014). Mastery of the local language allows students to better understand cultural nuances, navigate daily life, and build relationships with classmates and faculty, contributing to a sense of belonging in the academic environment (Wang and Hannes, 2014; Yang et al., 2006). This sense of belonging can positively influence motivation and persistence, both of which are essential for academic achievement. Additionally, developing L2 skills equips students with critical thinking and problem-solving abilities as they learn to interpret and convey complex ideas in a new linguistic context. These cognitive skills not only improve academic outcomes but also prepare students for success in an increasingly globalized professional landscape. Hence, L2 language study is indispensable for academic success and personal growth when studying abroad.

### 1.2 The present study: context and rationale

To frame the relationship between L2 proficiency and academic performance, this study draws on Cummins (1979) theory of Cognitive Academic Language Proficiency (CALP), which emphasizes the role of advanced language skills in supporting academic achievement. In the context of Chinese-medium education, HSK measures multiple dimensions of L2 ability, including listening, reading, and writing. These components align with core academic tasks such as attending lectures, reading scholarly texts, and producing written assignments. This study, therefore, considers how individual HSK components may differentially predict academic success, contributing to a more nuanced understanding of language readiness among international students.

For international students studying in China, proficiency in Mandarin, as measured by HSK, allows students to participate actively in discussions, comprehend complex academic texts, and follow lectures, which are often conducted entirely in Chinese. Moreover, language proficiency impacts students’ ability to access local networks and support systems (Meng et al., 2018; Rui and Wang, 2015; Hirai et al., 2015), which can contribute to academic and personal well-being. Effective L2 acquisition can bridge the gap between academic potential and actual performance by enabling students to grasp content, articulate insights, and complete academic tasks with greater ease.

While there is a substantial body of research examining the impact of L2 skills on academic performance, especially for students studying in English-speaking countries, the literature specifically addressing the academic experiences of students in China and the role of HSK proficiency remains limited. A few studies have documented positive associations between HSK scores and academic performance in Chinese-medium programs, though these findings primarily pertain to the undergraduate level rather than graduate education. For example, a correlation analysis conducted at Beijing Language and Culture University with 30 international students found that HSK Level 4 scores were significantly associated with course outcomes (Kong and Zhang, 2024). In a four-year degree program, Liu and Moody (2025) reported that HSK progression closely aligned with broader communicative competence, suggesting its potential relevance for academic success. Collectively, these findings support the validity of HSK as an indicator of learners’ readiness for academic study in Chinese-medium contexts.

However, the potential benefits of higher HSK scores on international students’ academic performance during their first year of graduate study in China remain largely unexplored. This study aims to address this gap. The HSK test provides separate scores for multiple language domains, including listening, reading, and writing. Understanding the intercorrelations among these subcomponents can offer insights into how different aspects of L2 proficiency co-vary. Such analysis contributes to the interpretation of HSK as a composite measure and helps clarify whether specific language skills are overlapping or distinct in the way they are assessed.

In this study, a robust Bayesian logistic modeling method is proposed to estimate the probability of an international student achieving a high letter grade in the first year of graduate school in China, based on the average undergraduate score, HSK listening, reading and writing scores. The robust Bayesian logistic model, rather than the standard Bayesian logistic model, is chosen given its benefit of minimizing the effect of extreme values on regression results (Kruschke, 2015). The proposed model is tested for a dataset comprised of 666 data points. This study introduces a nuanced perspective that synergizes quantitative analysis with practical, actionable insights, on the L2 language skills on students’ academic performance.

### 1.3 Research questions

Based on the identified research gaps concerning the role of L2 proficiency in academic performance among international students in Chinese-medium graduate programs, the following research questions (RQ) are proposed:

*RQ1*: What are the intercorrelations among the component scores of the HSK test (e.g., listening, reading, writing)?

*RQ2*: How are individual HSK component scores associated with the probability of achieving a final coursework grade above 85 during the first year of graduate study?

*RQ3*: How accurately can a predictive model, based on HSK component scores and undergraduate academic performance, estimate the likelihood of achieving academic success during the first year of graduate study?

## 2 Materials and methods

### 2.1 Data

To conduct a comprehensive study, data were collected from a few universities that are mainly in south and central-south China. The selection of participating institutions was based on the criterion that each university had a relatively large population of international students (i.e., more than 100), which ensured an adequate sample size for meaningful analysis. After identifying eligible universities, the research team submitted formal data access requests to the respective graduate schools. Upon receiving institutional approval, the research team contacted individual international students to explain the purpose of the study and to request their consent for data access. With informed consent, students’ HSK scores and their first-year graduate coursework grades were retrieved from official academic records maintained by the graduate schools.

While these institutions offer certain programs in English, all students included in this study were enrolled in graduate programs conducted entirely in Chinese. Participants were primarily majoring in science, engineering, or medical disciplines. Students in Chinese language and literature programs were excluded from the dataset, as their advanced Mandarin proficiency would not be comparable to students in non-language disciplines. This sampling approach ensured that all participants were engaged in content-based coursework taught in Mandarin, making L2 proficiency a relevant factor for academic performance. The dataset spans three academic years—2017–2018, 2018–2019, and 2023–2024—and comprises data from 666 students across the three universities mentioned above. The years 2020–2022 were excluded due to the limited number of students during the COVID-19 pandemic.

Two additional pieces of information were collected for this study. The first is the students’ undergraduate academic performance, reported as an average score on a 100-point scale. Most students’ scores ranged from 75 to 90. The second is their academic performance during their first year of graduate school. Focusing on the first year was strategic for two reasons. First, the study aims to explore the potential impact of L2 (second language) proficiency on academic achievement. The HSK score reflects students’ Chinese language proficiency at the start of their graduate studies. As students continue their education and live in a Mandarin-speaking environment, their language skills typically improve, making the original HSK score less accurate by the second year. Therefore, we focus on first-year academic performance, which aligns more closely with the timing of the HSK score.

For analysis, the first-year graduate school scores were converted to letter grades, with an average score of 85 equivalent to a B+. A binary variable was introduced to indicate whether a student achieved an average score of at least 85 (binary variable = 1) or less than 85 (binary variable = 0). This range (85–100) is typically classified as “Excellent” and is widely used to denote high academic achievement. It is also frequently applied as a benchmark for scholarship qualification or course progression, particularly at the graduate level. While this threshold serves as a reference point for categorization, it is important to note that the analytical method employed in this study is not dependent on the specific value chosen. The model is robust to changes in threshold criteria and can accommodate alternative cutoffs if institutional standards vary.

An independent samples t-test showed no significant difference in HSK scores between male and female students (*p* > 0.05); therefore, gender was not included as a control variable in subsequent analyses. Due to limited data availability, information on prior experience in China (e.g., duration of residence or previous education) could not be comprehensively analyzed and is acknowledged as a limitation of the study.

### 2.2 Robust Bayesian logistic modeling

**Figure 1:**
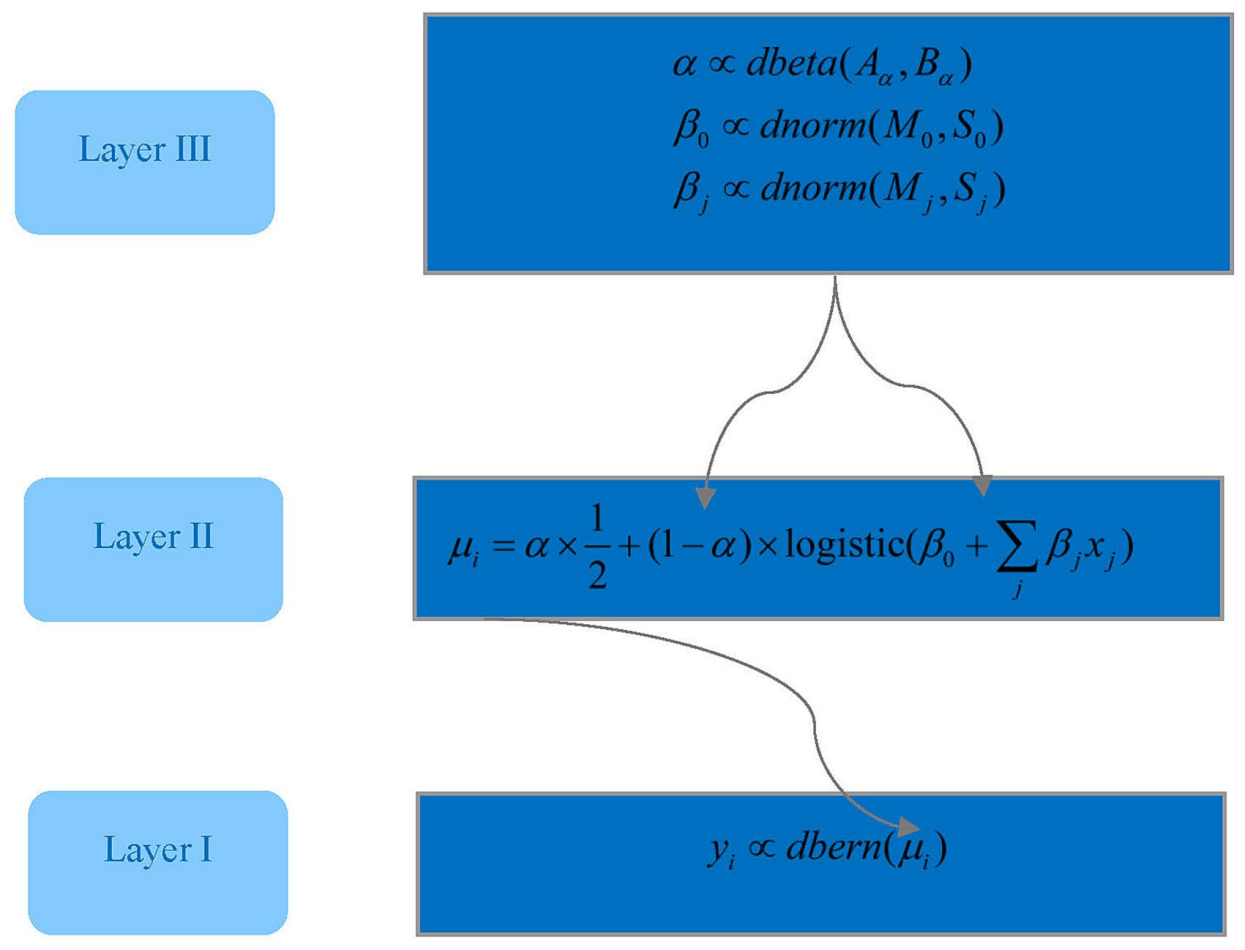
The three-layer structure of the logistic Bayesian Model used in this study.

The fundamental principle of the logistic regression model lies in constructing a linear combination of selected predictors, which is then converted into a probability using the logistic function. However, a key limitation of logistic regression is its susceptibility to overlooking outliers in the dataset. To mitigate this limitation, an additional parameter has been introduced in our analysis. Figure 1 illustrates the structural framework of the robust Bayesian logistic regression model, emphasizing the hierarchical structure inherent in the proposed approach.

The notations and expressions in Figure 1 represent the model parameters and their corresponding probability distribution functions. The variable *y_i_* denotes a binary outcome. For example, a value of 1 is assigned to *y_i_* in instances where the student’s grade in the first year in graduate school is B+ and above; otherwise, it is assigned as 0. The random variable *y_i_* is assumed to follow a Bernoulli distribution, defined by the parameter *μ_i_* as shown in Equation 1 and depicted in the model structure in Figure 1. The parameter *μ_i_* is further specified in Equation 2.

The first term in Equation 2 is a “guessing” parameter, representing the probability that the binary outcome (0 or 1) results from a Bernoulli random process with a parameter value of 0.5. The second term incorporates a linear combination of predictors, such as the HSK listening score and reading score, along with intercept parameters *β*_0_ and slope parameters *β_j_*, where *j* indexes the set of predictors. This formulation allows for the accommodation of outliers, addressing anomalies that are not adequately captured by the established relationship between predictors and the outcome variable.

In practical terms, Equation 2 can be understood as a weighted blend of a predefined random process—specifically, a Bernoulli process with a parameter value of 0.5—and a logistic function. When the “guessing” parameter is set to zero, the equation simplifies into a standard logistic function. Conversely, maximizing the “guessing” parameter modifies Equation 2 to represent a purely random Bernoulli process.


(1) $$y_{i}\sim \text{Bernoulli}(\mu_{i})$$

(2) $$\mu_{i}=\alpha \times \frac{1}{2}+(1-\alpha )\times \text{logistic}(\beta_{0}+\sum_{j}\beta_{j}x_{j,i})$$

(3) $$\beta_{0}\sim N(M_{0},S_{0})$$

(4) $$\beta_{j}\sim N(M_{j},S_{j})$$

(5) α~dbeta(1,100)

In total, there are five parameters in the hierarchical Bayesian model, including α , $\beta_{0}$ , $\beta_{1}$ , $\beta_{2}$ , $\;\beta_{3}$ , and $\beta_{4}$ , when HSK listening, reading, and writing scores, as well as averaged undergraduate score, are used in the logistic function. Prior distributions of the model parameters are shown in Equations 3–5. Specifically, the guessing parameter is modeled using a Beta distribution with a small mean value. This prior distribution is chosen to assign greater weight to the logistic function. Meanwhile, the intercept parameter $\beta_{0}$ and four slope parameters are assumed to follow the normal distribution.

The posterior distribution of the model parameters was estimated using the Markov Chain Monte Carlo (MCMC) method with the Gibbs sampling algorithm. This process was implemented through the “Just Another Gibbs Sampler” (JAGS) package, using four independent chains. To improve the efficiency of the MCMC algorithm, a preprocessing step was performed in which predictors were standardized. Specifically, HSK score and averaged undergraduate score were standardized prior to being incorporated into the modeling process (Kruschke, 2015).

A Bayesian logistic regression model was chosen for three reasons. First, unlike classical logistic regression, the Bayesian framework yields full posterior distributions of coefficients, enabling more comprehensive quantification of uncertainty through credible intervals. Second, the robust Bayesian formulation incorporates a “guessing” parameter, which helps reduce the influence of outliers, an advantage over standard logistic regression. Third, Bayesian modeling provides interpretable parameter estimates, allowing us to directly examine the academic significance of Chinese language proficiency relative to prior academic performance. Alternative classification algorithms such as random forest or support vector machines could also be applied, but these methods focus primarily on prediction and offer less interpretability in terms of variable contributions. Since the purpose of this study is explanatory rather than purely predictive, Bayesian logistic regression is most appropriate.

### 2.3 Evaluation metrics

Several evaluation metrics were utilized in this study to evaluate the quality of probabilistic categorical forecasts. The primary metric is the Brier Score (BS), which measures the mean squared error of the forecasts. The formula for calculating the Brier Score is presented in Equation 6.


(6) $$\mathrm{BS}=\frac{1}{J}\sum_{j=1}^{J}{\left(p_{j}-o_{j}\right)}^{2}$$

where $p_{j}$ is the predictive probability of a student’s letter grade being B+ and above in the first year in graduate school and $o_{j}$ refers to the observed binary value. In this study, $o_{j}$ is 1 if the student’s letter grade is B+ and above and it is zero if the student’s letter grade in graduate program is below B+. The predictive probability is calculated based on the model described in the last section. Notation *J* represents the total number of students used for model validation.

The second evaluation metric used in this study is the Brier Skill Score (BSS), which facilitates a comparative assessment of the Brier Score from the proposed model against a reference forecasting approach. In this context, the reference model is defined as the baseline probability of achieving a B+ or above, calculated from the observed dataset comprising 666 data points. The mathematical formulation for computing the BSS is provided in Equation 7. The Brier Skill Score ranges from −∞ to 1, where a score of 1 indicates perfect predictive accuracy and a clear improvement over the reference model. A score of 0 implies performance equivalent to the reference, while negative values suggest that the model performs worse than the baseline forecast.


(7) $$\mathrm{BSS}=1-\frac{\mathrm{BS}}{{\mathrm{BS}}_{0}}$$

This study also considers two additional metrics: the true positive rate (TPR) and the false positive rate (FPR). The TPR measures the proportion of instances where the model correctly predicts the occurrence of a binary event, while the FPR quantifies the proportion of cases where the model incorrectly predicts an event that does not occur. For instance, an FPR might arise when the model predicts that a student will achieve a letter grade of B+ or higher, but the student fails to do so. The mathematical formulas for calculating the TPR and FPR are presented in Equations 8, 9, respectively.


(8) $$\mathrm{TP}=\frac{n}{m}$$

(9) $$\mathrm{FP}=\frac{v}{w}$$

In Equation 8, the variable *m* denotes the total number of students who achieved a letter grade of B+ or higher. Among these *m* students, *n* represents the number of cases in which the predictive model correctly forecasted a B+ or higher outcome. Similarly, in Equation 9, the variable *w* denotes the total number of students who did not achieve a B+ or higher. Within this group, *v* represents the number of cases in which the model incorrectly predicted a B+ or higher grade.

## 3 Results

### 3.1 Descriptive statistics

**Figure 2:**
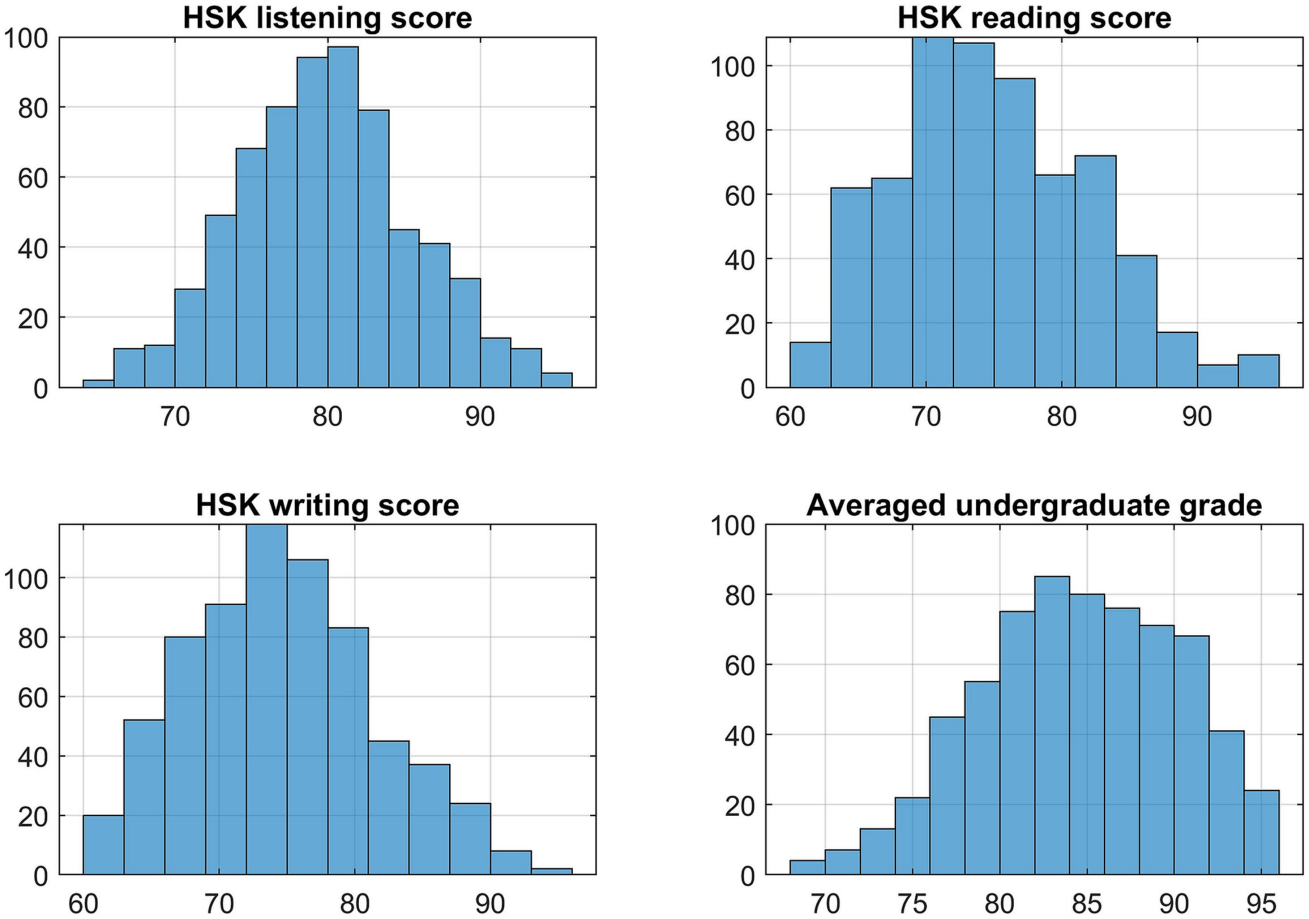
The histograms of HSK scores and average undergraduate scores for the 666 students included in the study.

Figure 2 presents histograms of HSK scores and average undergraduate scores for the 666 students included in the study. Several observations can be drawn from the data. Among the three HSK test components, listening scores are the highest on average, with a mean of 79.8. In contrast, the average scores for reading and writing are nearly identical, both at 75. For 48.35% of the students (322 out of 666), listening scores exceed 80, whereas only 25.2% exceed 80 in reading and 21.2% in writing. Regarding undergraduate academic performance, approximately 78% of students (520 out of 666) have average scores above 80, and 48% (322 out of 666) score above 85. Additional descriptive statistics are summarized in Table 1.

**Table 1:** Descriptive statistics of averaged undergraduate score and HSK scores.

Predictor	Mean	Standard deviation	25th–75th percentiles
Undergraduate score	84.6	5.69	80.5–89.2
Listening	79.8	5.74	76.0–83.0
Reading	75.0	7.12	70.0–80.0
Writing	74.6	6.91	69.0–79.0

The HSK listening scores also exhibit a smaller standard deviation compared to reading and writing scores, indicating less variability. This is further reflected in its narrower interquartile range (IQR), defined as the difference between the 25th and 75th percentiles, compared to the IQRs for reading and writing scores.

**Figure 3:**
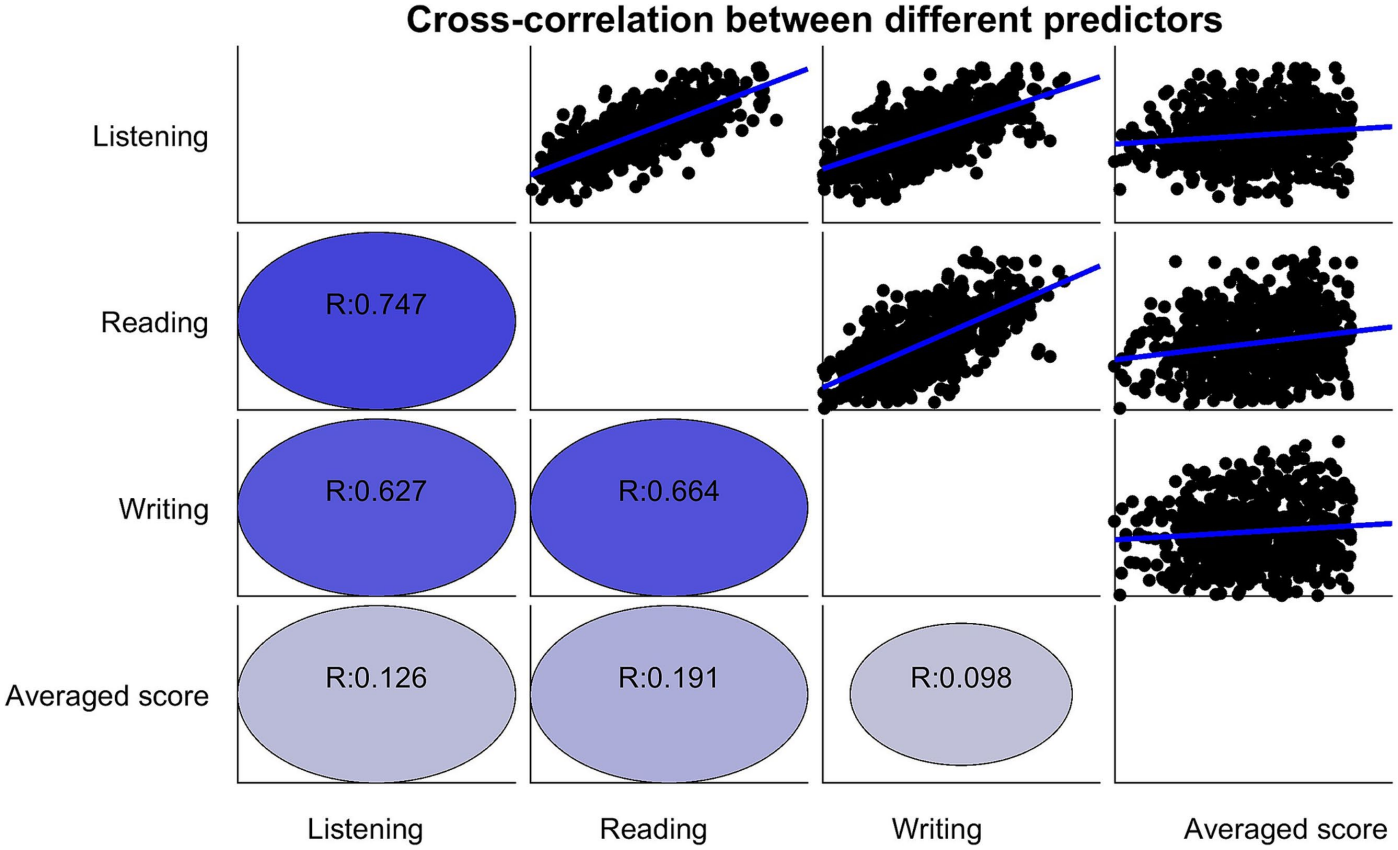
The cross-correlation between different predictors.

Figure 3 illustrates the strong cross-correlation among the HSK scores. The Pearson correlation coefficient between HSK listening and reading scores for the 666 students is approximately 0.75, which is statistically significant at the 0.05 level. This high correlation suggests that both skills rely heavily on common underlying factors, such as L2 vocabulary knowledge. Similarly, the Pearson correlation coefficient between listening and writing scores is 0.63, while the correlation between reading and writing scores is 0.67, further indicating strong interdependencies among these skills. The computed Variance Inflation Factors (VIFs) for the predictors HSK listening, HSK reading, HSK writing, and Undergraduate GPA is 2.74, 2.85, 2.67, and 1.12, respectively. These VIF values are below the conventional threshold of 5, suggesting that while multicollinearity is present among the HSK subskills, it is not at a level that invalidates the model presented in this study.

In contrast, the correlations between HSK scores and the average undergraduate score are much weaker, ranging from 0.01 to 0.13. While HSK reading and listening scores show slightly higher correlations with the undergraduate score, these values are not practically significant. This suggests that L2 test scores are largely independent of academic performance, which may be more influenced by the individual effort students invest in L2 language learning and standardized test preparation.

**Figure 4:**
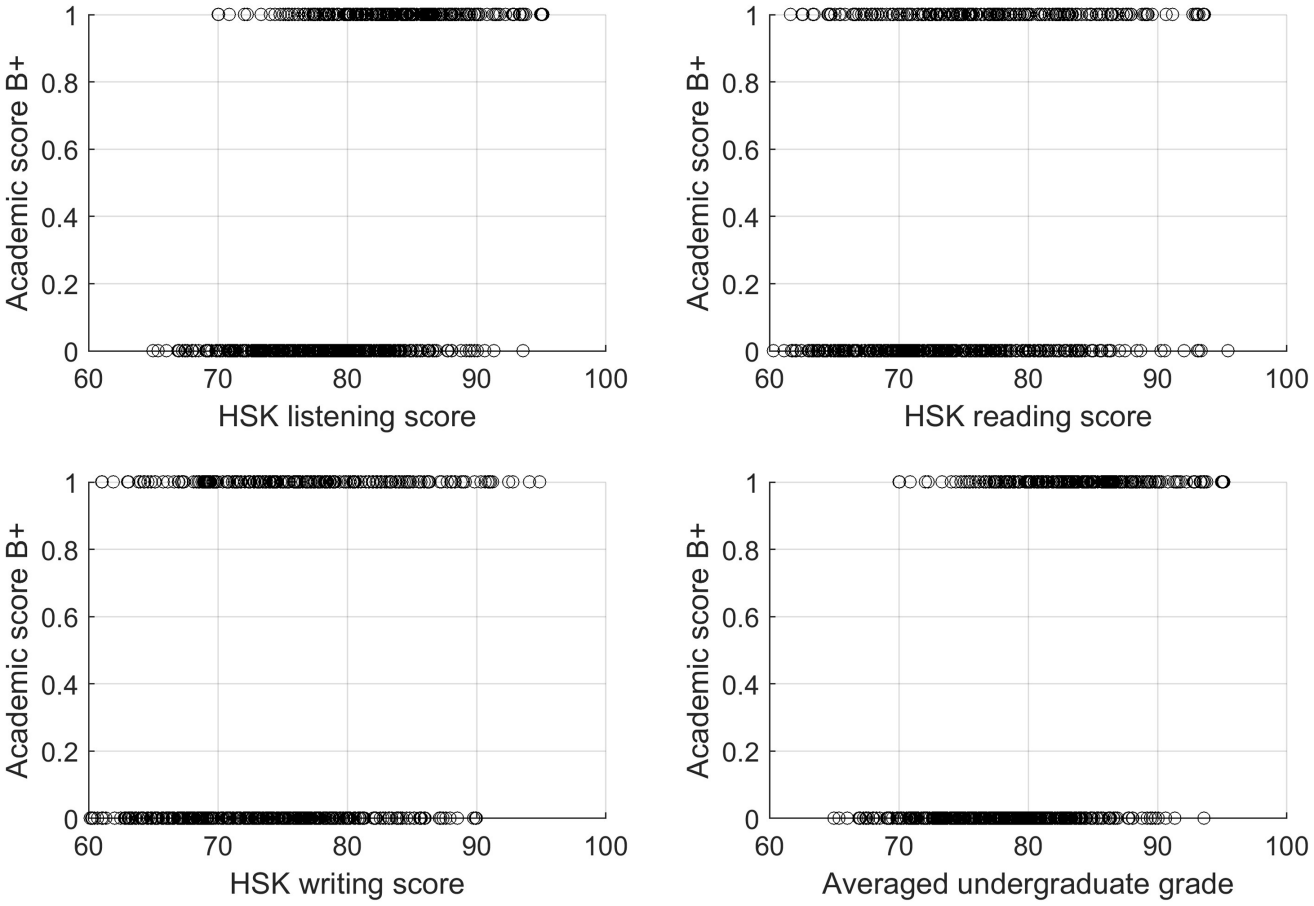
The scatterplots between academic score B+, a binary variable, and potential predictors.

Figure 4 presents scatterplots illustrating the relationship between first-year graduate academic performance and its four predictors. As described earlier, academic performance is represented as a binary variable, with a value of 1 indicating an average score of 85 or higher (B+ or above), and 0 otherwise. Several insights can be drawn from this figure. The likelihood of achieving a B+ grade appears to increase with higher HSK scores, even though the exact impact of these scores is difficult to quantify without a predictive model. For example, among the students with HSK listening scores above 90, 86% (25 out of 29) achieved a B+. Similarly, 59% (10 out of 17) of those with HSK reading scores above 90 achieved a B+. In contrast, lower HSK scores are associated with a reduced likelihood of obtaining a B+ grade. Specifically, when HSK listening scores fall below 70, the chances of achieving a B+ grade diminish significantly. The average undergraduate score shows a stronger relationship with academic performance in graduate school compared to HSK scores. Students with an average undergraduate score of 85 or higher achieved a B+ grade in 67.7% (218 out of 322) of cases. Conversely, only 7.85% (27 out of 344) of students with an undergraduate score below 85 achieved a B+ grade and above.

### 3.2 Bayesian logistic modeling for academic performance

In deriving and evaluating the Bayesian logistic model, 80% of the data was used for estimating model parameters and the remaining 20% was reserved for model evaluation. We adopted an 80/20 train-test split, with 80% of the dataset used for parameter estimation and 20% reserved for independent validation. This choice was guided by two considerations. First, the dataset size (*n* = 666) provided sufficient cases in both subsets to ensure stable estimation and meaningful evaluation. Second, given the computational intensity of Bayesian MCMC sampling, repeated cross-validation was not feasible without substantially increasing computational burden. The 80/20 split also has the advantage of providing a fully independent validation set, which is often considered a rigorous standard in predictive modeling.

**Figure 5:**
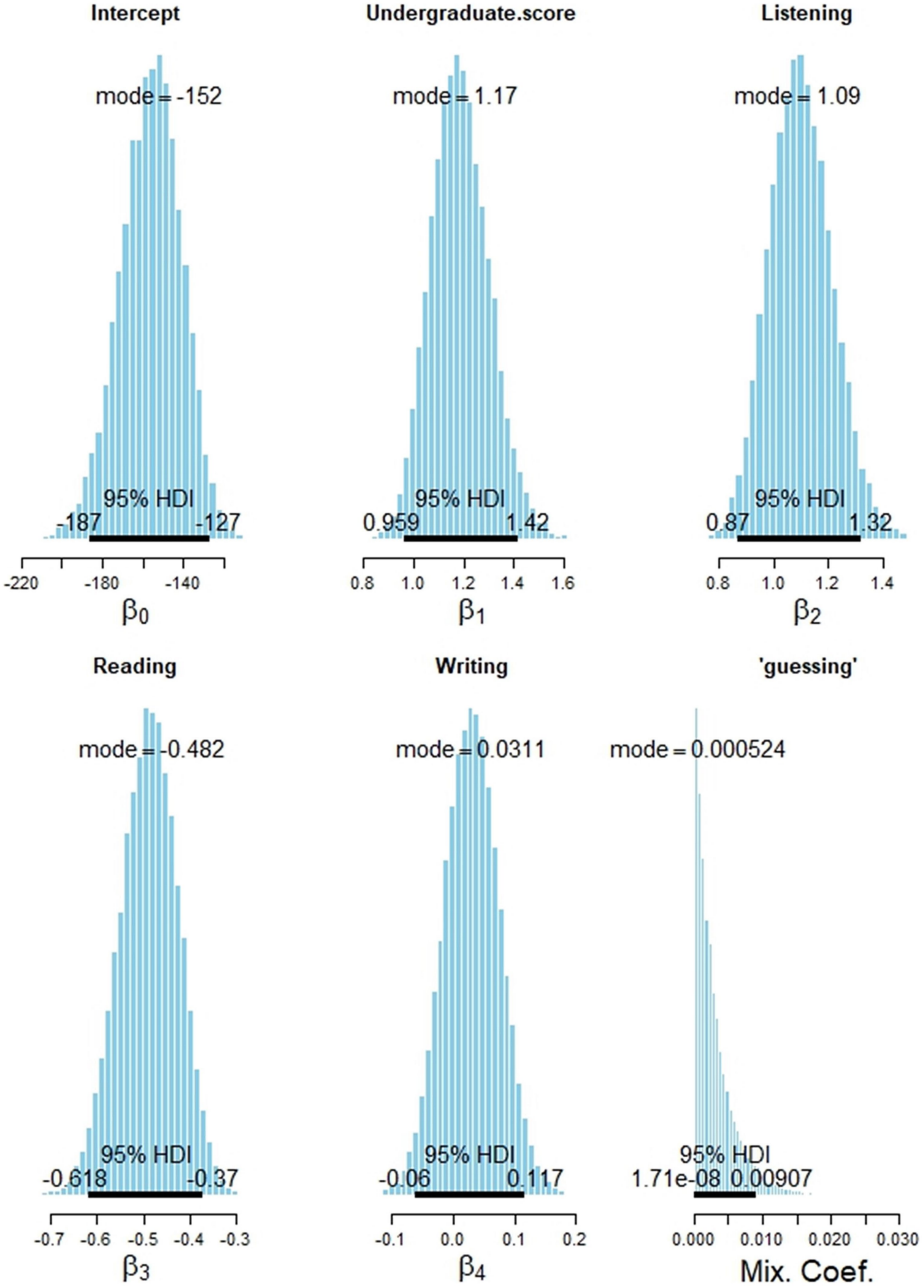
Posterior distribution of the model parameters of the fitted logistic Bayesian model.

Figure 5 shows the estimated posterior distributions of model parameters, including the mode and the 95% high-density interval (HDI). The mode of the intercept parameter is −152; the 95% HDI ranges from −187 to −127. The mode of the coefficient parameter associated with undergraduate score is 1.17; the 95% HDI ranges from 0.96 to 1.42. The mode of the coefficient parameter associated with HSK listening, reading, and writing is 1.09, −0.482, and 0.0311, respectively. The model of the guessing parameter is close to zero, indicating the chance of assigning to a random student a high graduate score of B+ and above is minimum without knowing the student’s information. It is, however, nonzero with the benefit of accommodating outliers in the dataset. The standardized coefficients are reported in the Supplementary material.

The estimated coefficients for each predictor represent their potential influence on the likelihood that an L2 student will achieve a B+ or higher in their first year of graduate school. Among all predictors, the average undergraduate score has the largest positive coefficient, suggesting it has the strongest impact on subsequent academic performance. Notably, while the coefficient for HSK listening is positive with a mode of 1.09, the coefficient for HSK reading is negative. This does not imply that stronger Chinese reading skills hinder academic performance. Rather, it reflects the multicollinearity among the three HSK components—listening, reading, and writing—which are strongly correlated. In a multivariate logistic regression model, the algorithm attempts to isolate the unique contribution of each predictor while accounting for the effects of the others. This process can result in a positive coefficient for one variable and a negative coefficient for another, despite their overall positive relationships with the outcome. Consistent with this, the coefficient for HSK reading is negative, while the coefficient for HSK writing is small (less than 0.05), indicating a minimal unique effect after adjusting for the other language components. The predictive performance of the Bayesian regression model is evaluated in the following section.

**Figure 6:**
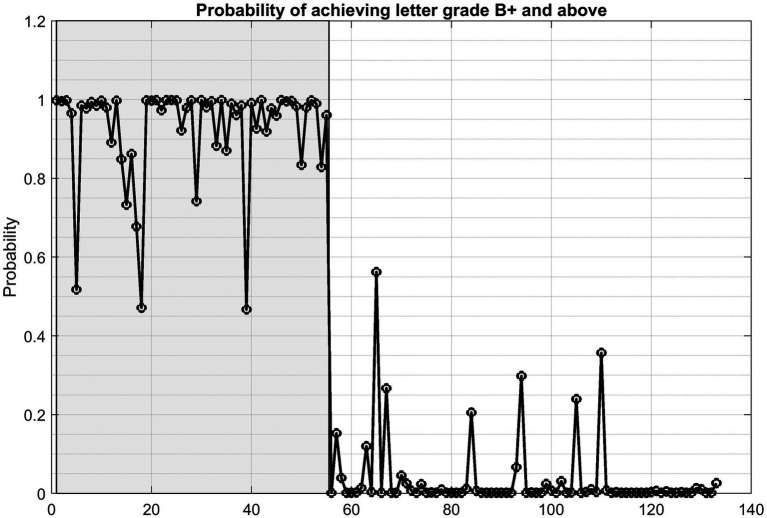
The mean predicted probability of achieving a B+ or higher letter grade in the first year of graduate study for the test dataset.

Figure 6 illustrates the mean predicted probability of achieving a B+ or higher letter grade in the first year of graduate study. The first 55 data points in the validation dataset, indicated within the shaded region, represent students who actually achieved a B+ or higher, while the remaining data points correspond to students with lower grades. This visual representation highlights a clear trend: the estimated probabilities are generally much lower for students who did not achieve a B+ or higher.

A probability threshold of 0.6 is applied in evaluating the model results. This slightly stricter cutoff than the conventional 0.50 was chosen for two reasons. First, in educational contexts, false positives are considered more costly than false negatives, and a higher threshold provides a more conservative decision rule. When a probability threshold of 0.6 is applied, 52 out of the 55 students with a B+ or higher have predicted probabilities exceeding the threshold. Conversely, among the 78 students with lower grades, none have predicted probabilities above 0.6. Only 3 of the 55 positive cases fall below the threshold, while all negative cases fall below it. As a result, the model achieves a true positive rate (TPR) of 0.945 (52/55) and a false positive rate (FPR) of 0 (0/78), indicating strong discriminatory power. At a classification threshold of 0.6, the confusion matrix indicated 52 true positives, 78 true negatives, 0 false positives, and 3 false negatives. This corresponds to an accuracy of 97.7%.

Compared to the baseline model—which assigns a uniform probability of 0.368 (the overall proportion of students achieving a B+ or higher in the dataset)—the proposed Bayesian logistic regression model demonstrates substantially better predictive performance. The Brier Score for the fitted model is 0.015, while the Brier Score for the baseline model is 0.25. This large difference indicates that the proposed model provides significantly more accurate probability estimates. Furthermore, the Brier Skill Score (BSS) is 0.94, confirming that the predictive performance of the proposed model far exceeds that of the baseline.

## 4 Discussion

This study aimed to explore the role of L2 proficiency in international students’ academic performance through three key research questions.

*RQ1*: What are the intercorrelations among the component scores of the HSK test (e.g., listening, reading, writing)?

The findings revealed strong positive correlations among HSK listening, reading, and writing scores. This suggests that these language skills are interdependent and may reflect a shared underlying competence, such as vocabulary knowledge or general language processing ability. These correlations support the idea that students who perform well in one language domain are likely to perform well in others, which is consistent with prior research on integrated L2 proficiency.

*RQ2*: How are individual HSK component scores associated with the probability of achieving a final coursework grade above 85 during the first year of graduate study?

While the analysis indicates that undergraduate academic performance is a stronger predictor of first-year graduate success than HSK scores, this does not diminish the important role that language proficiency plays in shaping international students’ academic outcomes. The programs in this study were all taught in Mandarin, and thus required students to engage with lectures, assignments, and assessments in a second language. Our findings show that higher HSK scores—particularly in reading and listening—are associated with greater likelihood of academic success, even if the effect size is smaller than that of prior academic performance.

While all three HSK components show some positive association with academic success, listening proficiency demonstrated the strongest predictive relationship. Reading and writing scores had more modest or mixed effects, likely due to multicollinearity among the three predictors. These findings suggest that L2 listening skills, which are critical for understanding lectures and class interactions, may be particularly important for academic success in Mandarin-medium graduate programs.

*RQ3*: How accurately can a predictive model, based on HSK component scores and undergraduate academic performance, estimate the likelihood of achieving academic success during the first year of graduate study?

The Bayesian logistic model performed well in estimating the likelihood of achieving high academic performance. The model’s predictive accuracy, supported by strong Brier and Brier Skill Scores, suggests that the combined use of L2 proficiency scores and prior academic performance yields reliable estimates of graduate outcomes. Notably, prior undergraduate academic performance emerged as the strongest predictor, reinforcing its foundational role.

Despite this, language skills should not be overlooked. In Chinese-medium programs, the ability to comprehend spoken and written Mandarin remains a necessary condition for academic engagement. Thus, while academic background may serve as a robust predictor, L2 proficiency continues to play a critical supporting role in students’ academic trajectories.

This study, while offering valuable insights into the predictors of academic performance among international graduate students in China, has several limitations that warrant consideration. First, the participating universities are primarily located in South and Central-South China. Although selection was based on the size of their international student populations rather than geographic location, this regional concentration may limit the generalizability of the findings. Institutional differences across regions—such as academic expectations, support systems, and instructional practices—could influence how language proficiency affects academic performance. Second, the countries or regions of origin of the international students may shape their language learning experiences, academic preparation, and adaptation to Chinese-medium instruction. Cultural, educational, and linguistic backgrounds may mediate the relationship between HSK proficiency and academic success. Future research should therefore include a more geographically and demographically diverse sample to validate and extend these findings. Finally, the study lacks detailed data on participants’ prior experience in China, such as earlier study or duration of residence. These factors may influence both language proficiency and academic adjustment. Including such variables in future studies would allow for a more nuanced examination of their potential moderating effects on academic performance.

## 5 Conclusion

This study contributes to our understanding of how language proficiency and prior academic performance jointly shape the academic success of international graduate students in Chinese-medium programs. By applying a robust Bayesian logistic model to a sample of 666 students, we have provided a nuanced picture of how HSK sub-scores and undergraduate GPA predict first-year academic achievement. This study extends existing L2 academic performance literature by shifting the geographical and linguistic context to China and the HSK test, which remains underexplored. Our findings contribute to theory by demonstrating that different components of L2 proficiency (especially listening) may play differentiated roles in academic success. This insight supports the relevance of frameworks like CALP (Cummins, 1979) in Chinese-medium educational environments. From a practical standpoint, the study suggests that university admissions policies should consider both undergraduate academic records and specific language skills—especially listening proficiency—when evaluating international applicants. Furthermore, language support programs that target listening skills may be particularly effective in supporting student success.

Future research should explore the evolving influence of L2 proficiency over the full duration of graduate programs and incorporate other relevant variables such as prior study in China, disciplinary differences, and psychological adaptation factors. A longitudinal design could also better capture the dynamic interaction between language development and academic achievement.

## Data Availability

The raw data supporting the conclusions of this article will be made available by the authors, without undue reservation.
