# Supplementary figures and images for: Examining the benefit of L2 language proficiency on academic performance using Bayesian logistic modeling
Source: Front Psychol. 2025 Oct 1;16:1613695. doi: 10.3389/fpsyg.2025.1613695 (PMC12521212; doi:10.3389/fpsyg.2025.1613695)

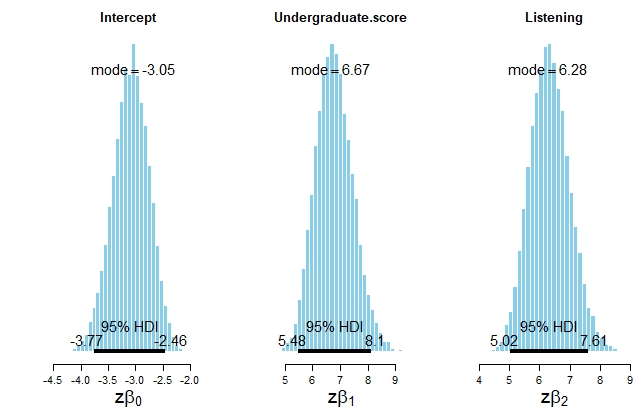

Supplement: Supplementary file 1 [file Image_1.JPEG]

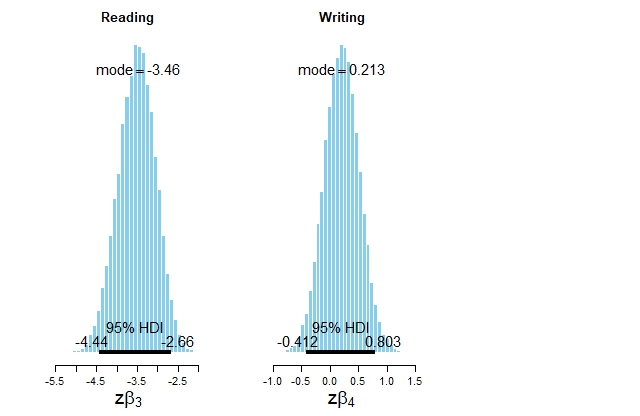

Supplement: Supplementary file 2 [file Image_2.JPEG]
